# Altered Neurometabolic Profile in Early Parkinson's Disease: A Study With Short Echo-Time Whole Brain MR Spectroscopic Imaging

**DOI:** 10.3389/fneur.2019.00777

**Published:** 2019-07-17

**Authors:** Martin Klietz, Paul Bronzlik, Patrick Nösel, Florian Wegner, Dirk W. Dressler, Mete Dadak, Andrew A. Maudsley, Sulaiman Sheriff, Heinrich Lanfermann, Xiao-Qi Ding

**Affiliations:** ^1^Department of Neurology, Hannover Medical School, Hanover, Germany; ^2^Department of Neuroradiology, Hannover Medical School, Hanover, Germany; ^3^Department of Radiology, University of Miami School of Medicine, Miami, FL, United States

**Keywords:** Parkinson's disease, whole brain, MRI, spectroscopy, biomarker, early diagnosis

## Abstract

**Objective:** To estimate alterations in neurometabolic profile of patients with early stage Parkinson's disease (PD) by using a short echo-time whole brain magnetic resonance spectroscopic imaging (wbMRSI) as possible biomarker for early diagnosis and monitoring of PD.

**Methods:** 20 PD patients in early stage (H&Y ≤ 2) without evidence of severe other diseases and 20 age and sex matched healthy controls underwent wbMRSI. In each subject brain regional concentrations of metabolites N-acetyl-aspartate (NAA), choline (Cho), total creatine (tCr), glutamine (Gln), glutamate (Glu), and myo-inositol (mIns) were obtained in atlas-defined lobar structures including subcortical basal ganglia structures (the left and right frontal lobes, temporal lobes, parietal lobes, occipital lobes, and the cerebellum) and compared between patients and matched healthy controls. Clinical characteristics of the PD patients were correlated with spectroscopic findings.

**Results:** In comparison to controls the PD patients revealed altered lobar metabolite levels in all brain lobes contralateral to dominantly affected body side, i.e., decreases of temporal NAA, Cho, and tCr, parietal NAA and tCr, and frontal as well as occipital NAA. The frontal NAA correlated negatively with the MDS-UPDRS II (*R* = 22120.585, *p* = 0.008), MDS-UPDRS IV (*R* = −0.458, *p* = 0.048) and total MDS-UPDRS scores (*R* = −0.679, *p* = 0.001).

**Conclusion:** In early PD stages metabolic alterations are evident in all contralateral brain lobes demonstrating that the neurodegenerative process affects not only local areas by dopaminergic denervation, but also the functional network within different brain regions. The wbMRSI-detectable brain metabolic alterations reveal the potential to serve as biomarkers for early PD.

## Introduction

Parkinson's disease (PD) is characterized by symptoms of rigidity, bradykinesia, tremor, and postural instability. Diagnosis is based on clinical findings, with an accuracy of only 53% for disease duration <5 years, increasing to 88% for durations longer than 5 years (1). Magnetic resonance imaging (MRI) reveals in PD patients only unspecific brain changes and is used mainly to exclude differential diagnoses (2–6). Magnetic resonance spectroscopy (MRS) can be used to measure brain metabolites like N-acetyl-aspartate (NAA), choline (Cho), myo-inositol (mIns), total creatine (tCr), glutamine (Gln), and glutamate (Glu), which provide information about neuronal integrity (NAA), membrane turnover (Cho), gliosis (mIns), energy metabolism (Cr), and glutamatergic neuronal activity (Glu, Gln) in patients. Numerous MRS studies on PD have been reported previously (7–11). Due to methodical limitations of commonly used MRS techniques that suffered from limited spatial coverage, most studies reported PD-related metabolic changes in one or a few small brain structures. These results thus may not necessarily reflect the metabolic status within whole brain. Considering that human brain functions as organized networks with interactions between different multiple brain regions (10, 12), information about PD-related metabolic alterations within the whole brain with high spatial resolution may help to better characterize PD and understand the underlying pathologic mechanisms. A recently established whole-brain MR spectroscopic imaging (wbMRSI) technique provides the possibility to measure brain metabolites simultaneously over different larger brain scales in subjects *in vivo* (13), as well as in multiple specific small brain areas (14, 15). Therefore, we are going to study altered brain metabolism in PD patients systematically by use of the wbMRSI. As a first part of the project we aimed to obtain an overview about altered neurometabolic profile in early PD by exploring metabolic changes in eight brain lobes and cerebellum that composed the whole brain, with the results being reported in the following.

## Patients and Methods

### Patients and Clinical Examinations

Human subject studies were carried out with approval from the local Ethics Committee of Hannover Medical School (No. 6167-2016) and all subjects gave written informed consent. PD patients were recruited from those treated at the neurological wards and movement disorders outpatient clinic of Hannover Medical School. Inclusion criteria were the neurological diagnosis of PD according to the Movement Disorder Society (MDS) diagnosis criteria with a Hoehn and Yahr stage (H&Y) of 1 or 2 in the best medical on state and age of 75 or below. Definition of early stage PD by the H&Y stage is in accordance with (7–13), additionally, none of our patients complained of a significant amount of motor complications qualifying for advanced PD [see (16) for review]. Patients with atypical Parkinsonism and other known brain pathologies e.g., stroke, small vessel disease or tumor, were excluded. Additionally, patients with severe head tremor, dystonia or dyskinesia had to be excluded from this study.

A movement disorders specialist enrolled the PD patients. Twenty PD patients (48–72 years old, mean age 60.2 ± 7.2 years, 8 males) were included. Information about course of PD in the individual patient was collected, including disease duration, dominantly affected body side, main symptoms, medication, and comorbidities. PD symptoms were assessed by the Movement Disorders Society Unified Parkinson's Disease Rating Scale (MDS-UPDRS) (17). Patients were rated in best medication “on” state. PD specific medication was noted and levodopa equivalence dosage calculated (LED). Cognitive deficits were quantified by the established test for dementia and mild cognitive impairment DemTect (18). As controls 20 healthy participants matched in age and sex on a one-to-one basis were also studied. All patients and healthy controls were right-handed according to self-report.

### MR Examinations

All subjects underwent MR examinations at 3T (Verio, Siemens, Erlangen, Germany). The routine MRI protocol included a T2 weighted turbo spin echo (TSE) sequence, a T2 weighted gradient echo (GRE) sequence, a fluid attenuation inversion recovery (FLAIR) sequence, a T1 weighted 3D magnetization-prepared rapid gradient-echo (MPRAGE) sequence, and a volumetric spin-echo planar spectroscopic imaging (EPSI) acquisition (TR/TE = 1550/17.6 ms, field-of view of 280 × 280 × 180 mm^3^, matrix size of 50 × 50 with 18 slices with a nominal voxel volume of 0.31 ml (= 5.6 × 5.6 × 10 mm^3^), echo train length of 1,000 points, and bandwidth of 2,500 Hz) for wbMRSI, as described previously (13, 19). The scan time with EPSI was about 17 min. The EPSI-acquisition included also a second dataset obtained without water suppression, which was used for several processing functions, including measurement and correction of the resonance frequency offset at each voxel location, correction of lineshape distortions and to provide internal signal reference for the normalization of metabolite concentrations (20), while the MPRAGE images were used as anatomical reference. The EPSI, MPRAGE, FLAIR, TSE, and GRE scans were obtained with the same angulation.

### Data Processing

**Figure 1:**
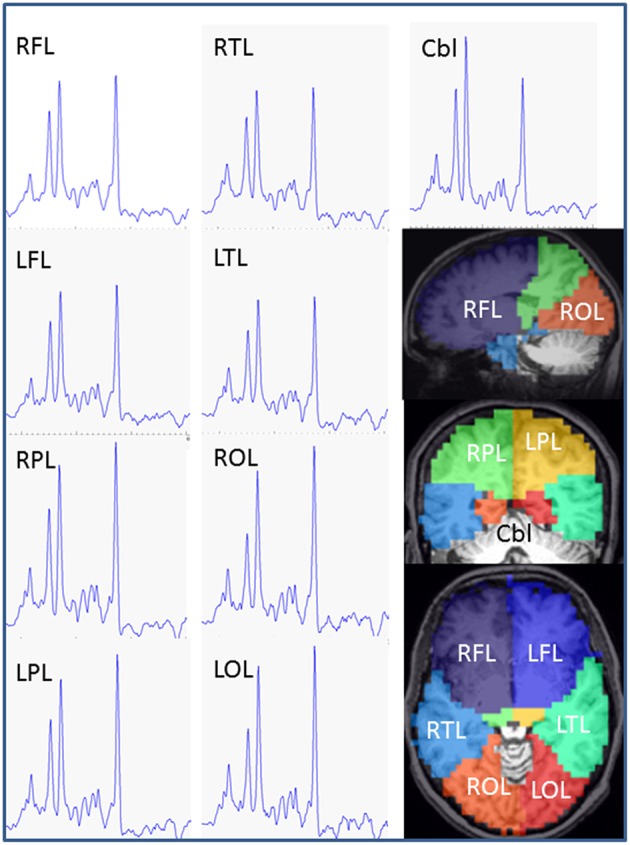
Exemplary MR spectra of each brain lobe and cerebellum obtained from a PD patient (female, 62 years). Estimated anatomic assignment of brain areas to brain lobes of our study. Frontal lobe: BA 4, 6, 8, 9, 10, 11, 12, 24, 25, 32, 33, 44, 45, 46, 47, head of caudate nucleus, accumbens, anterior part of putamen and pallidum, anterior cingulum. Parietal lobe: BA 1, 2, 3, 5, 7, 23, 31, 39, 40, thalamus, subthalamic nucleus, posterior cingulum. Temporal lobe: BA 13, 14, 15, 16, 20, 21, 22, 26, 27, 28, 29, 30, 34, 35, 36, 37, 38, 41, 42, 43, hippocampus, posterior parts of putamen and pallidum, caudatus tail, amygdala. Occipital lobe: BA 17, 18, 19. RFL, right frontal lobe; LFL, left frontal lobe; RPL, right parietal lobe; LPL, left parietal lobe; RTL, right temporal lobe; LTL left temporal lobe; ROL right occipital lobe; LOL left occipital lobe; Cbl, cerebellum; BA, Broadman Area.

MPRAGE, FLAIR, TSE, and GRE images were inspected to recognize possible morphological abnormalities, which were done by two neuroradiologists. The EPSI data were processed using the MIDAS software package to obtain volumetric metabolite maps. Processing included zero-filling to 64 × 64 × 32 points and spatial smoothing, resulting in an interpolated basic voxel volume of 0.107 ml (4.375 × 4.375 × 5.625 mm^3^) and an effective voxel volume of 1.5 ml (13, 19). The processing also included calculation of the fractional tissue volume contributing to each MRSI voxel, which used a tissue segmentation (18, 19) of the T1-weighted MPRAGE data to map gray matter (GM), white matter (WM), and cerebrospinal fluid (CSF). All resultant maps were then spatially transformed and interpolated to a standard spatial reference (20) at 2 mm isotropic resolution, which was associated with an atlas that mapped the individual brain lobes and the cerebellum. Mean regional metabolite concentrations were then determined in atlas-defined brain lobes and cerebellum, which composed the whole brain (Figure 1): The frontal lobe left (LFL) and right (RFL) including anterior parts of the striatum and pallidum, the temporal lobe left (LTL) and right (RTL) including posterior parts of the striatum and pallidum, the parietal lobe left (LPL) and right (RPL) including thalamus and subthalamic nucleus, the occipital lobe left (LOL) and right (ROL), and the cerebellum (Cbl). To obtain brain regional metabolite concentrations, especially to estimate the metabolites with small MRS signal amplitudes (Glu and Gln) separately, a modified data analysis approach suggested by Goryawala et al. was applied, i.e., the spectra were averaged by summing voxels within a region of interest (ROI) to obtain high-SNR spectra from atlas-registered anatomic regions, which was done following inverse spatial transformation of the atlas into subject space (14). Prior to averaging, the voxels were excluded if they had a spectral linewidth larger than 12 Hz or a CSF fraction larger than 30%. The application of these selection criteria resulted in excluding more basic voxels in frontal lobes (57% of the voxels within the structure), temporal lobes (55%), and cerebellum (54%) than in parietal (47%) and occipital lobes (33%), due to more filed distortion by neighbored structures containing bone and air or locations containing more CSF spaces. Finally, there were altogether 6,442 of 13,534 basic voxel spectra accounted for the spectral averaging in nine brain regions. The averaged spectra were subsequently analyzed with the FITT program included in MIDAS, in which a Lorentz-Gauss lineshape was used for spectral fitting. Mean regional concentrations of NAA, Cho, Cr, Glu, Gln, and mIns were determined as a ratio to a signal equivalent to that from 100% tissue water and presented as institutional units (i.u.) (21). Cramer-Rao lower bound (CRLB) of the spectral analysis was used as quality criteria for estimated metabolite values, i.e., only metabolites estimated with a CRLB <30% for Gln [a larger CRLB was selected for Gln to minimize possible bias related to its lower concentration (14)] and <20% for all other metabolites as often recommended for MRS analysis (http://s-provencher.com/lcmodel.shtml) were considered for further analyses. The fractional tissue volumes of CSF (FVCSF) and total brain tissue (FVBT) in each brain region were derived by using multi-voxel analysis based on nine atlas-defined anatomical regions. Correction for CSF volume contribution was applied as Met' = Met/(1-FVCSF).

### Statistical Analysis

The results from the patient studies were compared with those of 20 age- and sex-matched healthy controls. The normality of the data was checked with Shapiro-Wilk test, where more than 80% of the data were normally distributed (*p* > 0.05). Paired *t*-test was used for comparison of measured metabolite concentrations, spectral linewidths, and the fractional volumes of CSF in each of nine brain regions between patients and healthy controls. Wilcoxon signed rank test was additionally used for the few non-normally distributed data, which revealed the results with the same significance levels as those derived by using paired *t*-test, i.e., this part of the data showed no significant changes in patients by both parametric and nonparametric estimation. For simplicity, only the results of paired *t*- tests are given. In addition, the lobar metabolite concentrations of the patients were pooled in two hemispheres according to dominantly affected body side (right side in 12 patients and left side in 7 patients, one patient was excluded from this analysis due to no obvious dominantly affected body side), i.e., the contralateral brain lobes as well as ipsilateral brain lobes in respect to the affected/more affected body side, and compared to those of the healthy controls by using paired *t*-test. In addition, a nonparametric Spearman's correlation test was used to estimate possible correlations between clinical MDS-UPDRS and pooled lobar metabolites concentrations in patients. Corrections for multiple comparisons or multiple correlation tests were performed by using the false-discovery rate (FDR) method, with the desired false-discovery rate to 0.05. Those results with *p-*values not significant after a FDR correction were considered as showing a tendency (if *p* < 0.05) or a weak tendency (*p* < 0.75) of corresponding alterations in patients. Statistical analyses were performed with SPSS version 23 (SPSS IBM, New York, U.S.A.).

## Results

### PD Patient Characteristics

The 20 early stage PD subjects were clinically diagnosed. None of the patients was suspected to suffer from atypical Parkinsonism or was cognitively impaired. All patients reported an obvious positive response on dopaminergic treatment and were examined in the best medical on state. As summarized in Table 1, the Hoehn and Yahr stage of the PD subjects was 2 or less with mean disease duration of 6 years. Twelve PD subjects (60%) showed right side dominant symptoms, seven (35%) left side, and one patient (5%) presented mainly non-motor symptoms with no dominantly affected body side. Of our 20 PD subjects 4 presented with an akinetic-rigid type (20%), 8 showed a tremor dominant type (40%) or an equivalence type (40%), respectively. All the early PD subjects revealed good cognitive functions measured by the DemTect with a mean score of 15.5 ± 2.9. The patients displayed a mean value of 7.7 points in MDS-UPDRS part I as non-motor aspects of daily living (SD 4.5, min 2, max 20), and 7.0 in MDS-UPDRS part II as motor aspects of daily living (SD 4.3, min 2, max 17). Motor deficits in the best medical on of our PD subjects in the MDS-UPDRS part III scored in mean 15.4 points (SD 7.5, min 5, and max 31). Using the MDS-UPDRS part IV as scale for motor complications we measured a mean score of 0.35 points (SD 1, min 0, max 4).

**Table 1:** Patient characteristic.

	**Mean**	**SD**	**Min**	**Max**
Sex 12 female, 8 male				
Dominant side 12 right, 7 left				
Type 8 ET, 8 TD, 4 AR				
H&Y stage	1.6	0.5	1	2
Disease duration	6.0	3.7	1	13
MDS-UPDRS part I	7.7	4.5	2	20
MDS-UPDRS part II	7.0	4.3	2	17
MDS-UPDRS part III	15.4	7.5	5	31
MDS-UPDRS part IV	0.4	1	0	4
DemTect	15.9	1.9	13	18
LED	770 mg	521 mg	0 mg	1600 mg

*H&Y, Hoehn and Yahr stage; MDS-UPDS, Movement Disorders Society Unified Parkinson's Disease Rating Scale; DemTect test for cognitive assessment; LED, Levodopa-equivalence dosage*.

#### Whole-Brain MR Spectroscopic Imaging

**Figure 2:**
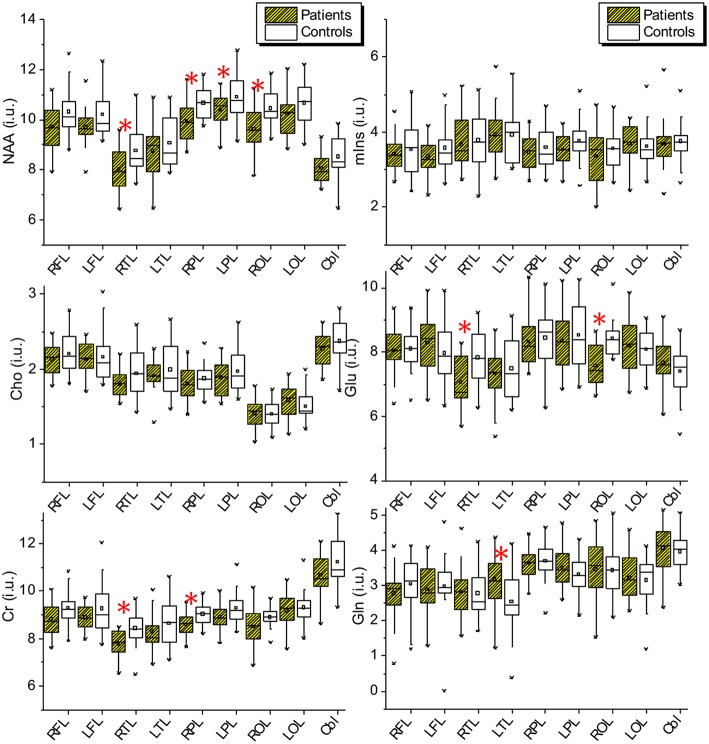
Lobar and cerebellar concentrations of NAA, Cho, tCr, Glu, Gln, and mIns measured in the PD patients and the healthy controls. NAA, N-acetyl-aspartat; mIns, myo-inusitol; Cho, choline; Glu, glutamate; Cr, creatinin; Gln, glumatine; RFL, right frontal lobe; LFL, left frontal lobe; RPL, right parietal lobe; LPL, left parietal lobe; RTL, right temporal lobe; LTL, left temporal lobe; ROL, right occipital lobe; LOL, left occipital lobe; Cbl, cerebellum.

Example averaged MR spectra of each brain lobe and cerebellum obtained from a PD patient (female, 62 years) are shown in Figure 1. The lobar and cerebellar concentrations of NAA, Cho, tCr, Glu, Gln, and mIns measured in the PD patients and healthy controls are drawn as Box-Whisker-plots in Figure 2, which shows that several lobes exhibit clear differences between the metabolite values of the patients and the controls.

Results of paired *t*-tests for comparisons of metabolite concentrations, spectral linewidths, and the fractional volumes of CSF and total brain tissue between patients and controls are shown in Tables 2, 3. Paired *t*-tests revealed that in patients NAA was decreased significantly in the right temporal lobe (−8.6%, *p* = 0.010), in the right parietal lobe (−6.7% and *p* = 0.007), and the right occipital lobe (−8%, *p* = 0.005), with a tendency to decrease in the left parietal lobe (*p* = 0.034) and with a weak tendency to decrease in both frontal lobes (*p* = 0.073 and 0.061 respectively); Cho did not show significant changes but revealed a weak tendency to decrease in right temporal lobe (*p* = 0.066); tCr was decreased significantly in the right temporal lobe (−7.6%, *p* = 0.004) and with a tendency to decrease in the right parietal lobe (−4.8%, *p* = 0.033); glutamate was decreased significantly in the right temporal lobe (−9.9%, *p* = 0.006) and the right occipital lobe (−10.2%, *p* = 0.001); glutamine showed a tendency to increase in the left temporal lobe (20.0%, *p* = 0.045); mIns did not show significant differences between patients and controls (Table 2). Moreover, in comparison to healthy controls the patients showed significantly broader spectral linewidths in both frontal lobes and the right occipital lobe, and with a trend to increase in the left occipital lobe. Slightly decreased FVCSF in frontal lobes and in left parietal lobe, and a slight increase of FVTB in left frontal lobe were also found in PD patients (Table 3). On the other hand, the cerebellum did not reveal significant differences between patients and controls concerning metabolite concentrations, spectral linewidth and FVCSF.

**Table 2:** Comparison of lobar and cerebellar metabolite concentrations between patients and controls with paired *t*-tests.

**Brain^**a**^**		**NAA (i.u.)**					**Cho (i.u.)**					**tCr (i.u.)**					
**Region**	***N*^**b**^**	**Patients**		**Controls**		***p***	**Patients**		**Controls**		***p***	**Patients**		**Controls**		***p***	
		**Mean**	**SD**	**Mean**	**SD**		**Mean**	**SD**	**Mean**	**SD**		**Mean**	**SD**	**Mean**	**SD**		
RFL	20	9.71	0.94	10.30	0.92	0.073	2.13	0.20	2.20	0.26	0.281	8.82	0.73	9.28	0.76	0.097	
LFL	20	9.69	0.74	10.20	0.93	0.061	2.13	0.22	2.16	0.34	0.735	8.89	0.52	9.26	1.04	0.186	
RTL	20	**8.00**	**0.83**	**8.75**	**0.96**	0.010^**^	1.80	0.18	1.94	0.31	0.066	**7.78**	**0.59**	**8.42**	**0.75**	0.004^**^	
LTL	20	8.73	1.07	9.06	1.07	0.382	1.91	0.22	1.99	0.35	0.374	8.29	0.83	8.63	0.99	0.343	
RPL	20	**9.94**	**0.82**	**10.66**	**0.66**	0.007^**^	1.81	0.22	1.87	0.20	0.343	**8.59**	**0.54**	**9.02**	**0.48**	0.033^*^	
LPL	20	**10.39**	**0.74**	**10.89**	**0.89**	0.034^*^	1.89	0.23	1.97	0.27	0.249	8.91	0.54	9.27	0.68	0.087	
ROL	20	**9.61**	**0.93**	**10.44**	**0.65**	0.005^**^	1.41	0.19	1.40	0.18	0.811	8.50	0.88	8.89	0.41	0.080	
LOL	20	10.23	0.92	10.66	0.89	0.155	1.58	0.23	1.51	0.21	0.198	9.16	0.78	9.31	0.71	0.544	
Cbl	20	8.06	0.64	8.50	0.86	0.127	2.27	0.23	2.37	0.29	0.249	10.66	0.81	11.23	0.98	0.087	
**Brain**		**mIns (i.u.)**					**Glu (i.u.)**					**Gln (i.u.)**					
**region**	***N***	**Patients**		**Controls**		***p***	**Patients**		**Controls**		***p***	***N***	**Patients**		**Controls**		***p***
		**Mean**	**SD**	**Mean**	**SD**		**Mean**	**SD**	**Mean**	**SD**			**Mean**	**SD**	**Mean**	**SD**	
RFL	20	3.42	0.48	3.54	0.71	0.573	8.04	0.82	8.10	0.67	0.791	16	3.04	0.50	3.20	0.66	0.403
LFL	20	3.34	0.44	3.57	0.65	0.250	8.27	0.97	7.96	0.90	0.339	16	3.05	0.61	3.15	0.57	0.672
RTL	20	3.66	0.68	3.78	0.77	0.595	**7.05**	**0.75**	**7.82**	**0.86**	0.006^**^	18	2.86	0.73	2.85	0.65	0.978
LTL	20	3.92	0.73	3.93	0.71	0.986	7.32	0.83	7.48	0.93	0.573	14	**3.34**	**0.63**	**2.78**	**0.65**	0.045^*^
RPL	20	3.46	0.49	3.58	0.61	0.442	8.32	0.78	8.43	0.91	0.638	20	3.63	0.45	3.69	0.53	0.694
LPL	20	3.54	0.44	3.76	0.56	0.188	8.35	0.94	8.52	1.07	0.555	20	3.51	0.55	3.32	0.51	0.142
ROL	20	3.36	0.67	3.56	0.55	0.211	**7.56**	**0.66**	**8.43**	**0.54**	0.001^**^	18	3.49	0.74	3.49	0.68	0.984
LOL	20	3.67	0.56	3.62	0.64	0.754	8.18	0.81	8.08	0.63	0.635	18	3.28	0.65	3.30	0.54	0.904
Cbl	20	3.67	0.65	3.75	0.57	0.671	7.67	0.73	7.39	0.76	0.201	19	4.14	0.69	3.90	0.54	0.325

a *Definition of brain regions: left and right frontal lobe (LFL/RFL), left and right temporal lobe (LTL/RTL), left and right parietal lobe (LPL/RPL), left and right occipital lobe (LOL/ROL), and cerebellum (Cbl)*.

b *Number of sampled subjects. Note that due to data quality controls (Crammer-Rao lower bond <30% for Gln, and <20% for all other metabolites) several subject pairs were not sampled for Gln analysis. Metabolites were determined as a ratio to a signal equivalent to that from 100% tissue water and presented as institutional unit (i.u.). SD = standard deviation. p-values <0.05 were presented in bold*.

** *Significant after correction for multiple comparisons by using false-discovery rate (FDR)*.

* *p < 0.05 but not significant after FDR correction*.

**Table 3:** Comparison of the spectral linewidths and the fractional volumes of cerebrospinal fluid and total brain tissue between patients and controls with paired *t*-tests.

**Brain^**a**^**		**Linewidth (Hz)**					**FVCSF**^****c****^					**FVTB**^****d****^				
**Region**		**Patients**		**Controls**		***p***	**Patients**		**Controls**		***p***	**Patients**		**Controls**		***p***
	***N*^**b**^**	**Mean**	**SD**	**Mean**	**SD**		**Mean**	**SD**	**Mean**	**SD**		**Mean**	**SD**	**Mean**	**SD**	
RFL	20	8.02	0.48	7.58	0.46	0.004^**^	0.079	0.006	0.084	0.008	0.024^*^	0.918	0.007	0.914	0.009	0.091
LFL	20	8.14	0.60	7.55	0.46	0.001^**^	0.079	0.008	0.086	0.008	0.000^**^	0.917	0.008	0.912	0.008	0.000^**^
RTL	20	8.01	0.73	7.90	0.54	0.540	0.075	0.019	0.074	0.014	0.786	0.923	0.018	0.923	0.014	0.849
LTL	20	7.91	0.73	7.64	0.51	0.201	0.082	0.016	0.082	0.015	0.983	0.916	0.016	0.916	0.015	0.961
RPL	20	7.26	0.58	7.11	0.57	0.272	0.091	0.012	0.096	0.014	0.069	0.902	0.016	0.899	0.015	0.406
LPL	20	7.24	0.51	7.05	0.56	0.187	0.090	0.011	0.096	0.013	0.040^*^	0.903	0.016	0.900	0.017	0.428
ROL	20	7.58	0.56	7.22	0.36	0.004^**^	0.067	0.020	0.065	0.016	0.557	0.928	0.020	0.931	0.016	0.518
LOL	20	7.55	0.72	7.19	0.29	0.034^*^	0.074	0.017	0.073	0.017	0.863	0.922	0.018	0.923	0.016	0.768
Cbl	20	8.27	1.09	8.04	0.64	0.425	0.063	0.012	0.065	0.010	0.615	0.935	0.012	0.933	0.011	0.679

a *Definition of brain regions: left and right frontal lobe (LFL/RFL), left and right temporal lobe (LTL/RTL), left and right parietal lobe (LPL/RPL), left and right occipital lobe (LOL/ROL), and cerebellum (Cbl)*.

b *Number of sampled subjects*.

c FVCSF represents the fractional volumes of cerebrospinal fluid.

d FVTB represents the fractional volumes of total brain tissue.

** *Significant after correction for multiple comparisons by using false-discovery rate (FDR)*.

* *p < 0.05 but not significant after FDR correction*.

The results of paired *t*-tests between pooled lobar metabolite concentrations of the patients and the controls are shown in Table 4, and those of the correlation tests of MDS-UPDRS to pooled lobar NAA concentrations in patients in Table 5. In comparison to healthy controls the patients revealed different grades of alterations in pooled lobar metabolite concentrations in respect to contralateral or ipsilateral hemispheres corresponding to the prominently affected body side.

**Table 4:** Paired *t*-test of lobar metabolite levels^a^ between patients and controls measured in brain hemisphere contralateral or ipsilateral to affected/more affected body side as indicated.

**Brain lobe**	**NAA (i.u.)**						**Cho (i.u.)**						**tCr (i.u.)**					
	***N*^**b**^**	**Patient**		**Control**		***p***	***N***	**Patient**		**Control**		***p***	***N***	**Patient**		**Control**		***p***
**Contralateral**		**Mean**	**SD**	**Mean**	**SD**			**Mean**	**SD**	**Mean**	**SD**			**Mean**	**SD**	**Mean**	**SD**	
Frontal	19	9.71	0.74	10.42	0.97	0.039^*^	19	2.13	0.20	2.21	0.33	0.368	19	8.88	0.69	9.41	1.01	*0.121*
Temporal	19	8.28	0.94	9.00	1.13	0.023^*^	19	1.84	0.20	2.01	0.31	0.030^*^	19	7.94	0.62	8.67	0.92	0.006^**^
Parietal	19	10.13	0.85	11.00	0.70	0.001^**^	19	1.83	0.25	1.95	0.25	0.083	19	8.68	0.53	9.27	0.64	0.009^**^
Occipital	19	9.87	0.95	10.50	0.85	0.049^*^	19	1.48	0.25	1.44	0.22	0.484	19	8.83	0.86	9.07	0.74	*0.319*
**Ipsilateral**																		
Frontal	19	9.87	0.76	10.11	0.88	0.340	19	2.17	0.19	2.15	0.28	0.824	19	8.91	0.55	9.09	0.79	0.386
Temporal	19	8.41	1.11	8.87	0.94	0.221	19	1.87	0.23	1.95	0.35	0.404	19	8.05	0.84	8.46	0.83	0.201
Parietal	19	10.33	0.69	10.69	0.76	0.154	19	1.89	0.21	1.91	0.22	0.821	19	8.86	0.59	9.07	0.53	0.295
Occipital	19	10.01	1.05	10.60	0.75	0.057	19	1.52	0.22	1.48	0.19	0.529	19	8.81	0.97	9.11	0.49	0.200
	**mIns (i.u.)**						**Glu (i.u.)**						**Gln (i.u.)**					
	***N***	**Patient**		**Control**		***p***	***N***	**Patient**		**Control**		***p***	***N***	**Patient**		**Control**		***p***
**Contralater**		**Mean**	**SD**	**Mean**	**SD**			**Mean**	**SD**	**Mean**	**SD**			**Mean**	**SD**	**Mean**	**SD**	
Frontal	19	3.40	0.49	3.74	0.67	0.128	19	8.22	0.82	8.07	0.86	0.638	14	3.28	0.47	3.13	0.60	0.501
Temporal	19	3.84	0.72	3.85	0.84	0.977	19	6.98	0.84	7.84	0.88	0.007^**^	15	3.10	0.82	2.89	0.65	0.517
Parietal	19	3.47	0.52	3.74	0.58	0.123	19	8.34	0.87	8.53	0.79	0.470	19	3.68	0.41	3.58	0.49	0.410
Occipital	19	3.51	0.60	3.55	0.67	0.836	19	7.94	0.69	8.03	0.52	0.670	17	3.45	0.66	3.54	0.69	0.586
**Ipsilateral**																		
Frontal	19	3.36	0.43	3.46	0.62	0.597	19	8.13	0.98	7.92	0.74	0.465	17	2.84	0.55	3.20	0.64	0.093
Temporal	19	3.65	0.58	3.93	0.65	0.225	19	7.29	0.71	7.46	0.92	0.518	15	3.06	0.67	2.69	0.62	0.127
Parietal	19	3.53	0.42	3.65	0.61	0.463	19	8.42	0.84	8.48	1.15	0.859	19	3.52	0.54	3.43	0.62	0.582
Occipital	19	3.55	0.68	3.63	0.55	0.584	19	7.91	0.87	8.46	0.65	0.059	17	3.28	0.71	3.33	0.47	0.800

a *Measured in ratio to brain internal water*.

b *Number of patient-control pairs. One patient was excluded from the analysis because of bilateral predominate symptoms*.

*Due to data quality criteria (Crammer-Rao lower bond less than 30% for Gln, and less than 20% for all other metabolites) data of several patients were not sampled for Gln by the analysis. Metabolites were determined as a ratio to a signal equivalent to that from 100% tissue water and presented as institutional unit (i.u.)*.

** *Significant after correction for multiple comparisons by using false-discovery rate (FDR)*.

* *p < 0.05 but not significant after FDR correction*.

**Table 5:** Correlations of MDS-UPDRS to brain NAA concentrations in respect to the most affected body side of the patients estimated by Spearman's correlation test^a^.

**Clinical scores**	**NAA in contralateral brain lobe**								
		**Frontal**		**Temporal**		**Parietal**		**Occipital**	
	***N***	***R***	***p***	***R***	***p***	***R***	***p***	***R***	***p***
UPDSR1	19	−0.359	0.131	−0.130	0.597	−0.034	0.892	0.055	0.824
UPDSR2	19	–**0.585**	0.008^**^	−0.050	0.840	−0.080	0.743	0.317	0.185
UPDSR3	19	−0.375	0.113	0.254	0.294	0.187	0.443	0.387	0.102
UPDRS4	19	**−0.458**	0.048^*^	−0.017	0.946	0.062	0.800	0.255	0.291
UPDRS	19	–**0.679**	0.001^**^	−0.055	0.822	−0.060	0.808	0.235	0.333
	**NAA in ipsilateral brain lobe**								
UPDSR1	19	0.184	0.450	−0.086	0.725	0.228	0.348	−0.030	0.903
UPDSR2	19	0.074	0.763	−0.233	0.338	0.000	1.000	−0.009	0.971
UPDSR3	19	0.211	0.386	−0.212	0.384	−0.062	0.800	−0.155	0.527
UPDRS4	19	−0.155	0.527	−0.338	0.157	−0.076	0.757	−0.035	0.888
UPDRS	19	0.208	0.392	−0.294	0.222	−0.034	0.889	−0.178	0.467

a *Twelve patients with more affected right body side and 7 with more affected left body side*.

** *Significant after correction for multiple comparisons by using false-discovery rate (FDR)*.

* *p < 0.05 but not significant after FDR correction. Bold values for results with p < 0.05*.

In the contralateral hemisphere, NAA decreased significantly or with a trend in all 4 brain lobes (−6.82% and *p* = 0.039 in FL, −8.00% and *p* = 0.023 in TL, −7.96% and *p* = 0.001 in PL, and −6.07% and *p* = 0.049 in OL); Cho showed a trend to decrease in one lobe (−8.47% and *p* = 0.030 in TL); tCr significantly decreased in two lobes (−8.52% and *p* = 0.006 in TL, and −6.40% and *p* = 0.009 in PL); glutamate decreased only in TL (−11.06% and *p* = 0.007); mIns and Gln did not show significant alterations (Table 4). Spearman's correlation test revealed significant correlations between MDS-UPDRS scores and lobar NAA concentrations in contralateral frontal lobe in patients, i.e., significant negative correlations of the NAA in contralateral frontal lobe to UPDRS2 as motor activities of daily living (*R* = −0.585, *p* = 0.008), and to total UPDRS (*R* = −0.679, *p* = 0.001) (Table 5), and a trend of negative correction to UPDRS4 as treatment complications (*R* = −0.458, *p* = 0.048).

In the ipsilateral hemisphere, only NAA and Glu revealed a weak tendency to decrease in occipital lobe (*p* = 0.057 for NAA and 0.059 for Glu) (Table 4), and no significant correlations of NAA to MDS -UPDRS was found (Table 5).

## Discussion

In this study we assessed changes of brain lobar and cerebellar metabolites in early stage PD. Our major findings are the significant alterations of NAA contents in PD subjects in the whole brain in comparison to age-matched healthy controls: NAA was decreased or showed a tendency to decreased values in a majority of brain lobes (6 of 8 lobes). Of course, variations in tissue water content, which was used to calibrate the metabolite contents, could impact the measured concentration of metabolites in spectroscopy, however, this would affect not only NAA but all metabolites and since they were not all altered significantly we found no evidence for changes in tissue water content. After pooling brain lobar metabolites in hemispheres contralateral and ipsilateral to the dominantly affected body side significantly decreased NAA contents was seen in all four contralateral brain lobes, where frontal lobar NAA revealed negative correlations to clinical scores of UPDRS2, UPDRS4, and total UPDRS. Moreover, we found tCr decreased in two contralateral brain lobes, and Cho and glutamate decreased each in one contralateral brain lobe. In parallel, a significant broadening of spectral linewidth was found in both frontal and occipital lobes.

The findings of decreased NAA, Cho, tCr, and Glu, and no changes of mIns were qualitatively consistent with those previously reported, despite several methodological differences on targeted brain regions or tissue type. For example, studies in *de novo* PD subjects showed a reduction in NAA in the motor cortex (22) and putamen (23). Decreased NAA/tCr and Glu/tCr in PD subjects with psychosis were reported (24). Brain NAA, Cho, and tCr were also measured over brain lobes with long echo time wbMRSI by Levin et al., who found decreased NAA/tCr and Cho/tCR in gray matter of temporal lobe, decreased NAA in right occipital lobe, and decreased NAA/tCr (25), anyhow, a detailed comparison to present study is difficult due to different patient selections and not separating tissue type between gray and white matter in the present. Since NAA is localized within neurons and involved in synaptic processes a decrease of brain NAA could be due to either a reduction in brain tissue volume or due to reduced neuronal function and metabolism (13). As no decrease of brain tissue volume in PD subjects was found the observed decreases of NAA most likely reflect reduced neuronal function and metabolism, which is consistent with the observations of decreased tCr and Glu, suggesting alterations in brain energy metabolism (tCr) and glutamatergic neuronal activity (Glu). The observed increase of glutamine in LTL could be a reactive response to reduced glutamate. Our findings of increased spectral linewidth in bilateral frontal and occipital lobes are most likely due to magnetic susceptibility-induced local magnetic field distortions and suggest pathological accumulation of brain iron in these brain regions, which has also been observed by other MRI measurements (26–28).

Present observation that brain lobar metabolite alterations were altered differently across the hemispheres contralateral and ipsilateral to the dominantly affected body side provides more insight into PD related brain metabolite changes. In the contralateral hemisphere, the main metabolic alterations were observed, which included significantly decreased NAA in all 4 brain lobes. In the ipsilateral hemisphere, smaller metabolic changes were seen, e.g., with PD subjects having lower mean NAA values in all lobes but not reaching significance. These observations indicate that the brain metabolite alterations are dominant in the hemisphere contralateral to more affected body side, reflecting PD-associated asymmetrical reduction of neuronal function and metabolism in early stage PD, which is consistent with previously reported lateralization findings of decreased NAA/tCr (29) and reduced dopamine uptake (30) in contralateral basal ganglia in early PD. Within the dominant hemisphere the distributions of the metabolic alterations varied among the brain lobes. The greatest changes occurred in the temporal lobe, with involvement of 3 metabolites (−8.00% for NAA, −8,47% for Cho, and −8.52% for tCr), the next occurred in the parietal lobe with involvement of 2 metabolites (−7.96% for NAA and −6.40% for tCr), while frontal and occipital lobes revealed decreases of one metabolite (−6.82% for FL and −6.07% for OL for NAA), showing the inhomogeneity of brain structures involved in PD process that may relate to their contained substructures. Interestingly, corresponding to the fact that the basal ganglia, which has been reported to be involved in PD pathological processes in previous MRS studies (22, 23, 31–36), are located in the temporal and frontal lobar atlas regions used in this study, we found that most metabolic changes occurred in temporal lobe, while the frontal NAA changes correlated significantly to MDS-UPDRS score describing clinical symptoms. Previous studies have reported a significant correlation of NAA/tCr to clinical symptoms (11, 31, 33, 34). However, this study found that tCr was also altered in PD, which is consistent with reduced concentrations of phosphates in the striatum and midbrain that has been interpreted as early mitochondrial dysfunction in PD patients (37). Therefore, the use of metabolite ratios to tCr may underestimate PD related metabolic changes.

This study performed the clinical evaluation and wbMRSI of the patients in their best medical condition; therefore, the low MDS-UPDRS part III score may underestimate the degree of disability as the treatment reduces this score by at least 20–30%. This may also have contributed to the lack of a significant correlation of the MDS-UPDRS III with spectroscopic findings. However, the MDS-UPDRS part II score for impairment of daily living reflects more selectively the impact of the disease (17, 24). The observed correlations of NAA in the frontal lobe, which includes important areas of the telencephalic dopaminergically innervated structures, with the MDS-UPDRS II, therefore, indicate the potential of wbMRSI for assessment of disability in Parkinson's disease (38, 39).

Treatment of PD is difficult and a lot of therapeutic agents are available (40). The impact of treatment in MRS studies has been rarely investigated. While some studies reported patients' on or off status during examination and imaging (11, 22, 25, 41–47), for a large number of studies the medication status is unclear (9, 31, 32, 37, 48–56). A recent study found significant metabolic changes in PD subjects between medical on and off state (41). These authors found a significant reduction in NAA, tCr, and mIns in the clinical off which is reversed for NAA und tCr under acute L-DOPA challenge (200 mg intake) (41). Another early PD spectroscopic study found no metabolic changes in the putamen before and after apomorphine therapy in 5 PD patients (57). Lucetti et al. reported an increased Cho/Cr ratio after 6 months of treatment with the dopamine agonist pergolide in early *de-novo* PD patients (43). Taking this limited amount of data together, PD therapy seems to impact spectroscopic measurements, however, dopamine agonists might not similarly influence spectroscopic changes of metabolite profile. Hence, the impact of different PD therapeutics is not yet clear and might vary in different brain regions. Dopaminergic innervated brain regions seem to show more likely PD medication dependent changes in metabolite profile. The short echo time wbMRSI offers a potential method to study these neurometabolic effects in more detail for a variety of brain regions in the future. Importantly, the impact of off state, acute levodopa challenge, and chronic treatment should be addressed in future studies.

It remains unclear which brain region and metabolites could be used as a valid spectroscopic marker for PD or atypical Parkinsonism. For this purpose wbMRSI could be very useful, because it provides a way to measure brain metabolites not only in large brain scales but also in multiple specific brain areas simultaneously, thus many hypotheses could be tested in one set of data (13, 19). Clinically established imaging diagnostics are often unspecific and in PD the MRI is often normal (2–6). In atypical Parkinsonism structural changes can be seen in MRI scans, e.g., changes like midbrain atrophy, putaminal rim, hot cross bun sign and others, are indicative of different atypical Parkinson syndromes, however, in the absence of these signs the diagnosis is difficult (4, 6, 58). DAT-SCAN as marker for degeneration of dopaminergic transporters on nigrostriatal projection neurons of the ventral midbrain is very sensitive for early detection of PD (59). The discrimination of Parkinson syndromes by the DAT-SCAN is not possible, because all syndromes have the common pathological hallmark of degeneration of midbrain dopaminergic neurons. By FDG-PET imaging PD and atypical Parkinsonism could be discriminated by specific metabolic patterns (60). Unfortunately, FDG-PET is off-label for the usage in the differential diagnosis of Parkinsonism. Furthermore, the methods of DAT-SCAN and FDG-PET require radiotracers. As an alternative WbMRSI offers a potential method for discrimination of PD and Parkinsonism without exposure to radioactive substances.

Present study focused on obtaining an overview of early PD-related metabolic alterations within the whole brain. Therefore, the cortical lobes and the cerebellum were selected as regions of interest in order to cover the whole brain. However, an accompanied limitation is that any regional metabolic inhomogeneity within the lobar or cerebellar structures was not accounted, especially the different contributions of the white matter, the cortical gray matter and basal ganglia were not separately evaluated. As an ongoing project PD related metabolic changes in multiple specific brain areas will be investigated in our further study. These results may then provide information related to specific brain functional networks and contribute to our understanding of the pathophysiological processes underlying PD.

Limitations of this study include the lack of correction of the results for age and for partial volume. However, the age effect was minimized by matching the patients and controls on a one-by-one basis appropriately in respect to age (and to gender). The partial volume effect was minimized by including only basic voxels containing <30% CSF for obtaining the integrated spectrum of each lobar brain region, and by correction for CSF volume contribution to measured metabolite values. Further validation of this study is also needed in a larger sample of patients together with a more comprehensive clinical phenotyping of PD subtypes (61–63). The identification of PD related metabolic changes in the white matter, cortical gray matter and basal ganglia may help to understand the metabolic processes during disease progression and spreading of neurodegenerative pathology in the brain (33, 64).

## Conclusion

This study has shown that NAA changed nearly ubiquitous in all brain lobes with different grades and with a clear lateralization contralateral to the major symptoms in early PD subjects. This finding suggests that NAA may be a promising spectroscopic marker for early diagnosis of PD (8), which is also favored by the observations of significant negative correlations between frontal NAA levels and the clinical UPDRS scores. Future studies with larger cohorts of patients with different stages of PD are needed to verify these results.

In conclusion, this study has demonstrated that even in early-stage PD brain metabolic alterations are evident and involved in all brain lobar areas of the cerebral hemisphere contralateral to the dominant side of disability. This result indicates that PD affects not only brain local regions by dopaminergic denervation, but also the brain network within the hemisphere. The novel wbMRSI-detectable brain metabolic alterations in PD may serve as promising biomarkers for early PD diagnosis, differential diagnosis of Parkinsonism (32) and with emerging disease-modifying drugs also for treatment monitoring (65, 66).

## Ethics Statement

Human subject studies were carried out with approval from the local Ethics Committee of Hannover Medical School (No. 6167-2016) and all subjects gave written informed consent.

## Author Contributions

XD designed the study. MK, DD, and FW were responsible for recruitment. MK and PB performed the clinical characterization of the patients. PB and PN performed the MRIs. XD performed the statistical analysis. MK, FW, DD, MD, AM, SS, HL, and XD interpreted the data. MK, FW, and XD wrote the manuscript. PB, DD, MD, AM, SS, and HL coedited the manuscript. All authors had access to the data generated in the study including the statistical analysis and agree to submit the paper for publication.

### Conflict of Interest Statement

The authors declare that the research was conducted in the absence of any commercial or financial relationships that could be construed as a potential conflict of interest.
